# *N*-Methyl­isosalsoline from *Hammada scoparia*

**DOI:** 10.1107/S160053680802477X

**Published:** 2008-08-06

**Authors:** Raoudha Mezghani Jarraya, Amira Bouaziz, Besma Hamdi, Abdelhamid Ben Salah, Mohamed Damak

**Affiliations:** a Laboratoire de Chimie des Substances Naturelles, Faculté des Sciences de Sfax, BP 1171, 3000 Sfax, Tunisia; b Laboratoire des Sciences de Materiaux et d’Environnement, Faculté des Sciences de Sfax, BP 1171, 3000 Sfax, Tunisia

## Abstract

The title compound (systematic name: 1,2-dimethyl-6-meth­oxy-1,2,3,4-tetra­hydro­isoquinolin-7-ol), C_12_H_17_NO_2_, is a major alkaloid isolated from *Hammada scoparia* leaves. It belongs to the isoquinoline family and it was characterized by NMR spectroscopy and X-ray crystallographic techniques. The absolute configuration could not be reliably determined. An intermolecular O—H⋯N hydrogen bond is present in the crystal structure.

## Related literature

For related literature on *Hammada scoparia* and isoquinoline alkaloids, see: Baker (1996 ▶); Benkrief *et al.* (1990 ▶); Carling & Sandberg (1970 ▶); El-Shazly & Wink (2003 ▶); El-Shazly *et al.* (2005 ▶); Iwasa *et al.* (2001 ▶); Jarraya & Damak (2001 ▶); Vetulani *et al.* (2001 ▶, 2003 ▶).
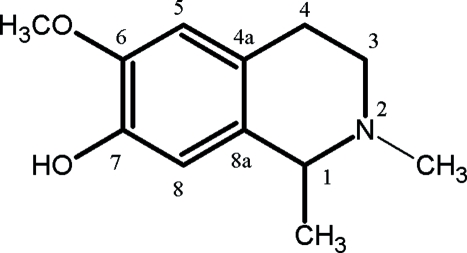


## Experimental

### 

#### Crystal data

C_12_H_17_NO_2_
*M* *_r_* = 207.27
Orthorhombic, 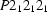

*a* = 7.5942 (6) Å
*b* = 10.8082 (8) Å
*c* = 13.2716 (10) Å
*V* = 1089.33 (14) Å^3^
*Z* = 4
Mo *K*α radiation
μ = 0.09 mm^−1^
*T* = 200 (2) K
0.48 × 0.37 × 0.22 mm

#### Data collection

Bruker SMART CCD area-detector diffractometer
Absorption correction: multi-scan (Becker & Coppens, 1974 ▶) *T*_min_ = 0.961, *T*_max_ = 0.988
23859 measured reflections
3132 independent reflections
2870 reflections with *I* > 2σ(*I*)
*R*_int_ = 0.030

#### Refinement

*R*[*F* ^2^ > 2σ(*F* ^2^)] = 0.033
*wR*(*F* ^2^) = 0.089
*S* = 1.06
3132 reflections
136 parameters
H-atom parameters constrained
Δρ_max_ = 0.44 e Å^−3^
Δρ_min_ = −0.24 e Å^−3^

### 

Data collection: *SMART* (Bruker, 1998 ▶); cell refinement: *SAINT* (Bruker, 1998 ▶); data reduction: *SAINT*; program(s) used to solve structure: *SHELXS97* (Sheldrick, 2008 ▶); program(s) used to refine structure: *SHELXL97* (Sheldrick, 2008 ▶); molecular graphics: *ORTEP-3* (Farrugia, 1997 ▶); software used to prepare material for publication: *WinGX* (Farrugia, 1999 ▶).

## Supplementary Material

Crystal structure: contains datablocks I, global. DOI: 10.1107/S160053680802477X/zl2131sup1.cif

Structure factors: contains datablocks I. DOI: 10.1107/S160053680802477X/zl2131Isup2.hkl

Additional supplementary materials:  crystallographic information; 3D view; checkCIF report

## Figures and Tables

**Table 1:** Hydrogen-bond geometry (Å, °)

*D*—H⋯*A*	*D*—H	H⋯*A*	*D*⋯*A*	*D*—H⋯*A*
O2—H2⋯N1^i^	0.84	1.90	2.6970 (10)	159

Symmetry code: (i) 
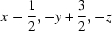
.

**Fig. 1.**
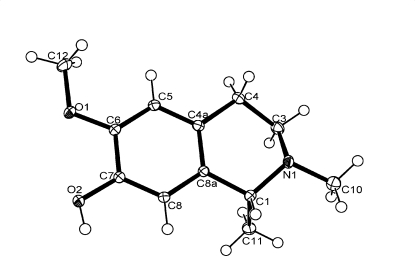
Molecular view of the title compound with the atom-labelling scheme. Ellispsoids are drawn at the 50% probability level. H atoms are represented as spheres of arbitrary radii.

**Fig. 2.**
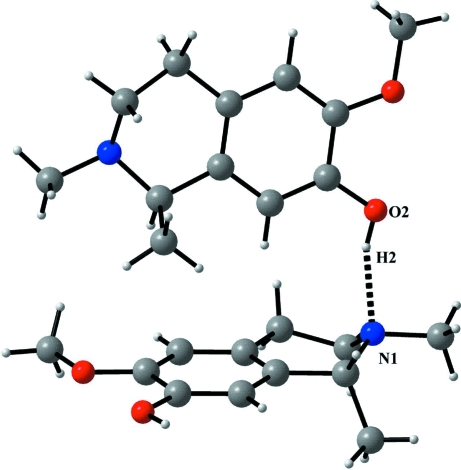
Partial packing view showing the formation of pseudo dimer through O—H···N hydrogen bonds. Hydrogen bonds are shown as dashed lines.

## supplementary crystallographic information

### Comment

*Hammada scoparia* has been reported to contain the alkaloids carnegine and *N*-methylisosalsoline as major tetrahydroisoquinoline alkaloids in addition to other minor alkaloids (Benkrief *et al.*, 1990, Jarraya & Damak 2001; El-Shazly & Wink, 2003).

The tetrahydroisoquinoline alkaloids affect the vegetative nervous system (Vetulani *et al.*, 2001 and 2003). Some of these alkaloids are known to be strong agonists at nicotinic acetylcholine receptors and it is thus likely that they serve as chemical defense compounds against insects and mammalian herbivores (El-Shazly *et al.*, 2005). Other simple isoquinoline alkaloids display potent, and often selective cytotoxicity or exhibit potential antimicrobial, antimalarial, antiviral and anti-HIV activities (Baker, 1996; Iwasa *et al.*, 2001).

The current study describes the isolation and the structure elucidation of *N*-methylisosalsoline. The structure of the title compound was established principally by two-dimensional NMR spectroscopy and through X-ray diffraction analysis although the absolute configuration could not be reliably determined.

The conformation of this compound is stabilized by an intermolecular hydrogen bond between the hydroxyl O_2_—H_2_ group and atom N_1_ (Table 1). The molecules are assembled by intermolecular O—H···N hydrogen bonds (Table 1, Fig. 2)

### Experimental

The title compound was extracted from *Hammada scoparia *leaves:

*Plant material Hammada scoparia* (Pomel) Iljin= (*Haloxylon scoparium* (Pomel) Bge. = *Haloxylon articulatum ssp scorparium* (Pomel) Batt. = *Arthrophytum scoparium* (Pomel) Iljin), belongs to Chenopodiaceae family and is locally known as "rimth" in Sfax, Tunisia.

Leaves were carefully detached from the fresh plant, collected in June 2007 in Sfax, Tunisia, and air-dried. Voucher specimens (LCSN101) have been deposited at the " Laboratoire de Chimie des Substances Naturelles", Faculty of Science, University of Sfax, Tunisia.

*Extraction and isolation of the N-Methylisosalsoline from Hammada scoparia leaves:*

Air-dried leaves of *Hammada scoparia* were extracted at room temperature during 48 h with a mixture (EtOH-H_2_O, 1–9, v-v). After filtration through folder filter paper Whatman N° 1, the ethanol was removed under reduced pressure and the remaining aqueous phase was acidified with HCl (pH = 3) and then defatted by extraction with CH_2_Cl_2_. The defatted mother liquor was made alkaline with an NH_4_OH solution (pH = 10) and immediately extracted with CH_2_Cl_2_ to exhaustion. The latter CH_2_Cl_2_ extract was concentrated to yield a reddish-brown residue (total alkaloids).

The total Alkaloids (5 g) were separated, on column chromatography over silica gel 60 (0.063–0.200 mm; 160 g), using a gradient of dichloromethane-methanol as eluents. Eleven fractions were isolated according to their similarity by thin layer chromatography analyses. Further purifications gave two major pure alkaloids; the first is oily: Carnegine (1050 mg; fraction 3 eluted with dichloromethane) and the second (white rosette crystals) is *N*-methylisosalsoline (545 mg; fraction 7 eluted with dichloromethane-methanol, 94–6, v-v). These alkaloids were previously isolated from *Hammada scoparia* (Carling & Sandberg, 1970; Benkrief *et al.*, 1990; Jarraya & Damak, 2001; El-Shazly & Wink, 2003). Their structures were determined on the basis of their spectral data such as UV, MS, ^1^H NMR and H—H COSY, ^13^C NMR (BB, DEPT and C—H COSY, HMQC, HMBC, NOESY) and confirmed by comparison with published spectra.

*N*-Methylisosalsoline (1-Methylcorypalline), white rosette crystals (MeOH), mp 443 K, UV λ_max_ (EtOH) nm = 207, 225, 285. λ_max_ (EtOH + OH^-^) nm = 213, 245, 300. EIMS, m/*z* (*rel*. int.): [*M*^+^] 207 (15), 193 (30), 192 (100), 177 (45), 164 (10), 149 (15), 121 (5), 96 (6), 91 (5), 77 (5), 57 (5), 42 (4). IR: (CHCl_3_) ν_max_ (cm^-1^): 3540, 2950, 2850, 2800, 1600, 1520.

Spectroscopic analysis, ^1^H NMR (300 MHz, CDCl_3_, p.p.m.): 1.34 (3*H*, d, J = 6.6 Hz, CH_3_–C_1_); 2.45 (3*H*, s, CH_3_–N); 2.63 (1*H*, ddd, J = 11.4, 6.9, 5.1 Hz, H–C_3_); 2.77 (2*H*, m, 2 H–C_4_); 3.02 (1*H*, ddd, J = 11.4, 6.9, 5.1 Hz,H–C_3_); 3.49 (1*H*, q, J = 6.6 Hz, H–C_1_); 3.83 (3*H*, s, CH_3_–O); 6.53 (1*H*,s, aromatic H, H–C_5_); 6.63 (1*H*, s, aromatic H, H–C_8_).

^13^C NMR (75.5 MHz, CDCl_3_, p.p.m.): 58.51, C_1_; 48.94, C_3 _; 27.36, C_4_; 124.84, C_4a_; 112.96, C_5_; 145.31, C_6_; 144.00, C_7_; 110.57, C_8_; 131.95, C_8a_; 19.45, CH_3_–C_1_; 42.70, CH_3_–N; 55.77, CH_3_–O. The HMQC spectra showed correlations between 112.96 (C_5_) and 6.53 (1*H*, s,aromatic H, H–C_5_); 110.57 (C_8_) and 6.63 (1*H*, s, aromatic H, H–C_8_).

Suitable white X-ray quality crystals of this compound were obtained by recrystallization from methanol.

### Refinement

All H atoms attached to C atoms and O atom were fixed geometrically and treated as riding with C—H = 0.98 Å (Cmethine), 0.97 Å (Cmethylene), 0.96Å (Cmethyl), 0.93Å (Caromatic) and O—H = 0.84 Å with *U*_iso_(H) = 1.2*U*_eq_(Cmethylene, Cmethine, Caromatic) or *U*_iso_(H) = 1.5*U*_eq_ (Cmethyl, O).

In the absence of significant anomalous scattering, the absolute configuration could not be reliably determined and then the Friedel pairs were merged and any references to the Flack parameter were removed.

### Crystal data

**Table:**

C_12_H_17_NO_2_	*D*_x_ = 1.264 Mg m^−^^3^
*M**_r_* = 207.27	Melting point: 473 K
Orthorhombic, *P*2_1_2_1_2_1_	Mo *K*α radiation λ = 0.71073 Å
Hall symbol: P 2ac 2ab	Cell parameters from 2100 reflections
*a* = 7.5942 (6) Å	θ = 2.7–21.3º
*b* = 10.8082 (8) Å	µ = 0.09 mm^−^^1^
*c* = 13.2716 (10) Å	*T* = 200 (2) K
*V* = 1089.33 (14) Å^3^	Prism, colourless
*Z* = 4	0.48 × 0.37 × 0.22 mm
*F*_000_ = 448	

### Data collection

**Table:**

Bruker SMART CCD area-detector diffractometer	3132 independent reflections
Radiation source: sealed tube	2870 reflections with *I* > 2σ(*I*)
Monochromator: graphite	*R*_int_ = 0.030
*T* = 200(2) K	θ_max_ = 37.1º
φ and ω scans	θ_min_ = 2.4º
Absorption correction: multi-scan(Becker & Coppens, 1974)	*h* = −12→10
*T*_min_ = 0.961, *T*_max_ = 0.988	*k* = −18→18
23859 measured reflections	*l* = −22→22

### Refinement

**Table:**

Refinement on *F*^2^	Secondary atom site location: difference Fourier map
Least-squares matrix: full	Hydrogen site location: inferred from neighbouring sites
*R*[*F*^2^ > 2σ(*F*^2^)] = 0.033	H-atom parameters constrained
*wR*(*F*^2^) = 0.089	*w* = 1/[σ^2^(*F*_o_^2^) + (0.0579*P*)^2^ + 0.028*P*] where *P* = (*F*_o_^2^ + 2*F*_c_^2^)/3
*S* = 1.07	(Δ/σ)_max_ = 0.001
3132 reflections	Δρ_max_ = 0.44 e Å^−^^3^
136 parameters	Δρ_min_ = −0.24 e Å^−^^3^
Primary atom site location: structure-invariant direct methods	Extinction correction: none

### Special details

**Table:**

Geometry. All e.s.d.'s (except the e.s.d. in the dihedral angle between two l.s. planes) are estimated using the full covariance matrix. The cell e.s.d.'s are taken into account individually in the estimation of e.s.d.'s in distances, angles and torsion angles; correlations between e.s.d.'s in cell parameters are only used when they are defined by crystal symmetry. An approximate (isotropic) treatment of cell e.s.d.'s is used for estimating e.s.d.'s involving l.s. planes.
Refinement. Refinement of *F*^2^ against ALL reflections. The weighted *R*-factor *wR* and goodness of fit *S* are based on *F*^2^, conventional *R*-factors *R* are based on *F*, with *F* set to zero for negative *F*^2^. The threshold expression of *F*^2^ > σ(*F*^2^) is used only for calculating *R*-factors(gt) *etc*. and is not relevant to the choice of reflections for refinement. *R*-factors based on *F*^2^ are statistically about twice as large as those based on *F*, and *R*- factors based on ALL data will be even larger.

### Fractional atomic coordinates and isotropic or equivalent isotropic displacement parameters (Å^2^)

**Table:**

	*x*	*y*	*z*	*U*_iso_*/*U*_eq_	
C1	0.05540 (10)	0.85207 (7)	0.13064 (6)	0.01206 (13)	
H1	0.0422	0.9013	0.0673	0.014*	
C3	0.25806 (11)	0.83001 (8)	0.27194 (6)	0.01586 (14)	
H3A	0.1557	0.8026	0.3124	0.019*	
H3B	0.3386	0.8766	0.3167	0.019*	
C4	0.35296 (11)	0.71807 (8)	0.22912 (6)	0.01475 (14)	
H4A	0.4694	0.7437	0.2029	0.018*	
H4B	0.3725	0.6567	0.2834	0.018*	
C4A	0.24733 (10)	0.65931 (7)	0.14534 (6)	0.01156 (13)	
C5	0.29165 (11)	0.54034 (7)	0.11057 (6)	0.01275 (13)	
H5	0.3867	0.4973	0.1412	0.015*	
C6	0.19977 (11)	0.48449 (7)	0.03258 (6)	0.01301 (13)	
C7	0.05722 (11)	0.54781 (7)	−0.01256 (6)	0.01242 (13)	
C8	0.01464 (10)	0.66497 (7)	0.02168 (6)	0.01194 (13)	
H8	−0.0812	0.7078	−0.0084	0.014*	
C8A	0.10928 (10)	0.72232 (7)	0.09969 (6)	0.01059 (12)	
C10	0.14695 (12)	1.03330 (8)	0.22797 (7)	0.01802 (15)	
H10A	0.1079	1.0854	0.1718	0.027*	
H10B	0.2483	1.0720	0.2611	0.027*	
H10C	0.0507	1.0242	0.2766	0.027*	
C11	−0.12539 (11)	0.85179 (8)	0.18297 (7)	0.01757 (15)	
H11A	−0.1569	0.9365	0.2022	0.026*	
H11B	−0.1200	0.7998	0.2434	0.026*	
H11C	−0.2144	0.8189	0.1367	0.026*	
C12	0.37900 (13)	0.30406 (9)	0.03586 (8)	0.02182 (18)	
H12A	0.3910	0.2239	0.0020	0.033*	
H12B	0.3579	0.2908	0.1079	0.033*	
H12C	0.4874	0.3520	0.0269	0.033*	
N1	0.19793 (9)	0.91062 (6)	0.18960 (5)	0.01301 (12)	
O1	0.23450 (9)	0.37016 (6)	−0.00665 (5)	0.01876 (13)	
O2	−0.02759 (9)	0.49009 (5)	−0.08915 (5)	0.01773 (13)	
H2	−0.1054	0.5372	−0.1126	0.027*	

### Atomic displacement parameters (Å^2^)

**Table:**

	*U*^11^	*U*^22^	*U*^33^	*U*^12^	*U*^13^	*U*^23^
C1	0.0121 (3)	0.0114 (3)	0.0127 (3)	0.0012 (2)	−0.0006 (2)	−0.0010 (2)
C3	0.0162 (3)	0.0185 (3)	0.0128 (3)	−0.0004 (3)	−0.0017 (3)	−0.0026 (3)
C4	0.0142 (3)	0.0154 (3)	0.0148 (3)	0.0000 (3)	−0.0041 (3)	−0.0009 (3)
C4A	0.0107 (3)	0.0121 (3)	0.0119 (3)	−0.0005 (2)	−0.0007 (2)	0.0007 (2)
C5	0.0119 (3)	0.0119 (3)	0.0144 (3)	0.0011 (2)	−0.0023 (2)	0.0009 (2)
C6	0.0132 (3)	0.0100 (3)	0.0159 (3)	0.0018 (2)	−0.0021 (3)	−0.0005 (2)
C7	0.0126 (3)	0.0102 (3)	0.0145 (3)	0.0007 (2)	−0.0027 (3)	−0.0004 (2)
C8	0.0116 (3)	0.0107 (3)	0.0135 (3)	0.0009 (2)	−0.0023 (2)	0.0000 (2)
C8A	0.0103 (3)	0.0100 (3)	0.0115 (3)	0.0001 (2)	−0.0002 (2)	0.0007 (2)
C10	0.0179 (4)	0.0149 (3)	0.0212 (3)	0.0006 (3)	0.0022 (3)	−0.0053 (3)
C11	0.0121 (3)	0.0191 (3)	0.0215 (3)	0.0012 (3)	0.0012 (3)	−0.0040 (3)
C12	0.0231 (4)	0.0173 (3)	0.0250 (4)	0.0099 (3)	−0.0070 (4)	−0.0022 (3)
N1	0.0132 (3)	0.0113 (3)	0.0145 (3)	−0.0006 (2)	0.0010 (2)	−0.0031 (2)
O1	0.0199 (3)	0.0113 (2)	0.0251 (3)	0.0056 (2)	−0.0085 (3)	−0.0043 (2)
O2	0.0189 (3)	0.0132 (2)	0.0211 (3)	0.0035 (2)	−0.0097 (2)	−0.0050 (2)

### Geometric parameters (Å, °)

**Table:**

C1—N1	1.4780 (10)	C7—O2	1.3555 (10)
C1—C8A	1.5174 (10)	C7—C8	1.3837 (10)
C1—C11	1.5386 (12)	C8—C8A	1.4045 (10)
C1—H1	1.0000	C8—H8	0.9500
C3—N1	1.4703 (11)	C10—N1	1.4722 (10)
C3—C4	1.5185 (12)	C10—H10A	0.9800
C3—H3A	0.9900	C10—H10B	0.9800
C3—H3B	0.9900	C10—H10C	0.9800
C4—C4A	1.5110 (11)	C11—H11A	0.9800
C4—H4A	0.9900	C11—H11B	0.9800
C4—H4B	0.9900	C11—H11C	0.9800
C4A—C8A	1.3892 (10)	C12—O1	1.4257 (11)
C4A—C5	1.4070 (11)	C12—H12A	0.9800
C5—C6	1.3866 (11)	C12—H12B	0.9800
C5—H5	0.9500	C12—H12C	0.9800
C6—O1	1.3665 (10)	O2—H2	0.8400
C6—C7	1.4139 (11)		
			
N1—C1—C8A	109.97 (6)	C7—C8—C8A	121.76 (7)
N1—C1—C11	114.55 (6)	C7—C8—H8	119.1
C8A—C1—C11	111.16 (7)	C8A—C8—H8	119.1
N1—C1—H1	106.9	C4A—C8A—C8	119.42 (7)
C8A—C1—H1	106.9	C4A—C8A—C1	122.58 (7)
C11—C1—H1	106.9	C8—C8A—C1	117.99 (7)
N1—C3—C4	109.96 (7)	N1—C10—H10A	109.5
N1—C3—H3A	109.7	N1—C10—H10B	109.5
C4—C3—H3A	109.7	H10A—C10—H10B	109.5
N1—C3—H3B	109.7	N1—C10—H10C	109.5
C4—C3—H3B	109.7	H10A—C10—H10C	109.5
H3A—C3—H3B	108.2	H10B—C10—H10C	109.5
C4A—C4—C3	110.99 (7)	C1—C11—H11A	109.5
C4A—C4—H4A	109.4	C1—C11—H11B	109.5
C3—C4—H4A	109.4	H11A—C11—H11B	109.5
C4A—C4—H4B	109.4	C1—C11—H11C	109.5
C3—C4—H4B	109.4	H11A—C11—H11C	109.5
H4A—C4—H4B	108.0	H11B—C11—H11C	109.5
C8A—C4A—C5	119.05 (7)	O1—C12—H12A	109.5
C8A—C4A—C4	121.03 (7)	O1—C12—H12B	109.5
C5—C4A—C4	119.90 (7)	H12A—C12—H12B	109.5
C6—C5—C4A	121.49 (7)	O1—C12—H12C	109.5
C6—C5—H5	119.3	H12A—C12—H12C	109.5
C4A—C5—H5	119.3	H12B—C12—H12C	109.5
O1—C6—C5	125.51 (7)	C3—N1—C10	111.00 (7)
O1—C6—C7	115.09 (7)	C3—N1—C1	111.54 (6)
C5—C6—C7	119.39 (7)	C10—N1—C1	112.10 (7)
O2—C7—C8	123.81 (7)	C6—O1—C12	116.80 (7)
O2—C7—C6	117.30 (7)	C7—O2—H2	109.5
C8—C7—C6	118.87 (7)		
			
N1—C3—C4—C4A	−48.19 (9)	C4—C4A—C8A—C1	−0.30 (11)
C3—C4—C4A—C8A	15.68 (11)	C7—C8—C8A—C4A	−1.26 (12)
C3—C4—C4A—C5	−166.13 (7)	C7—C8—C8A—C1	178.70 (7)
C8A—C4A—C5—C6	−0.56 (12)	N1—C1—C8A—C4A	16.96 (10)
C4—C4A—C5—C6	−178.78 (7)	C11—C1—C8A—C4A	−110.97 (8)
C4A—C5—C6—O1	179.31 (8)	N1—C1—C8A—C8	−163.01 (7)
C4A—C5—C6—C7	−0.60 (12)	C11—C1—C8A—C8	69.06 (9)
O1—C6—C7—O2	−0.54 (11)	C4—C3—N1—C10	−165.48 (7)
C5—C6—C7—O2	179.38 (7)	C4—C3—N1—C1	68.74 (8)
O1—C6—C7—C8	−179.10 (7)	C8A—C1—N1—C3	−50.54 (8)
C5—C6—C7—C8	0.82 (12)	C11—C1—N1—C3	75.49 (8)
O2—C7—C8—C8A	−178.36 (8)	C8A—C1—N1—C10	−175.71 (6)
C6—C7—C8—C8A	0.10 (12)	C11—C1—N1—C10	−49.68 (9)
C5—C4A—C8A—C8	1.47 (11)	C5—C6—O1—C12	−0.82 (13)
C4—C4A—C8A—C8	179.67 (7)	C7—C6—O1—C12	179.10 (8)
C5—C4A—C8A—C1	−178.50 (7)		

### Hydrogen-bond geometry (Å, °)

**Table:**

*D*—H···*A*	*D*—H	H···*A*	*D*···*A*	*D*—H···*A*
O2—H2···N1^i^	0.84	1.90	2.6970 (10)	159

Symmetry codes: (i) *x*−1/2, −*y*+3/2, −*z*.
